# Population structure and genetic diversity of 25 Russian sheep breeds based on whole-genome genotyping

**DOI:** 10.1186/s12711-018-0399-5

**Published:** 2018-05-24

**Authors:** Tatiana E. Deniskova, Arsen V. Dotsev, Marina I. Selionova, Elisabeth Kunz, Ivica Medugorac, Henry Reyer, Klaus Wimmers, Mario Barbato, Alexei A. Traspov, Gottfried Brem, Natalia A. Zinovieva

**Affiliations:** 1 L.K. Ernst Federal Science Center for Animal Husbandry, Dubrovitzy Estate 60, Podolia, Russia 142132; 2 All-Russian Research Institute of Sheep and Goat Breeding, Zootechnichesky Lane 15, Stavropol, Russia 355017; 3 Population Genomics Group, Department of Veterinary Sciences, LMU Munich, Veterinaerstr. 13, 80539 Munich, Germany; 4 Institute of Genome Biology, Leibniz Institute for Farm Animal Biology (FBN), Wilhelm-Stahl-Allee 2, 18196 Dummerstorf, Germany; 5 Istituto di Zootecnica, Università Cattolica del Sacro Cuore, Via Emilia Parmense 84, 29122 Piacenza, Italy; 6 Institute of Animal Breeding and Genetics, University of Veterinary Medicine Vienna, Veterinaerplatz 1, 1210 Vienna, Austria

## Abstract

**Background:**

Russia has a diverse variety of native and locally developed sheep breeds with coarse, fine, and semi-fine wool, which inhabit different climate zones and landscapes that range from hot deserts to harsh northern areas. To date, no genome-wide information has been used to investigate the history and genetic characteristics of the extant local Russian sheep populations. To infer the population structure and genome-wide diversity of Russian sheep, 25 local breeds were genotyped with the OvineSNP50 BeadChip. Furthermore, to evaluate admixture contributions from foreign breeds in Russian sheep, a set of 58 worldwide breeds from publicly available genotypes was added to our data.

**Results:**

We recorded similar observed heterozygosity (0.354–0.395) and allelic richness (1.890–1.955) levels across the analyzed breeds and they are comparable with those observed in the worldwide breeds. Recent effective population sizes estimated from linkage disequilibrium five generations ago ranged from 65 to 543. Multi-dimensional scaling, admixture, and neighbor-net analyses consistently identified a two-step subdivision of the Russian local sheep breeds. A first split clustered the Russian sheep populations according to their wool type (fine wool, semi-fine wool and coarse wool). The Dagestan Mountain and Baikal fine-fleeced breeds differ from the other Merino-derived local breeds. The semi-fine wool cluster combined a breed of Romanian origin, Tsigai, with its derivative Altai Mountain, the two Romney-introgressed breeds Kuibyshev and North Caucasian, and the Lincoln-introgressed Russian longhaired breed. The coarse-wool group comprised the Nordic short-tailed Romanov, the long-fat-tailed outlier Kuchugur and two clusters of fat-tailed sheep: the Caucasian Mountain breeds and the Buubei, Karakul, Edilbai, Kalmyk and Tuva breeds. The Russian fat-tailed breeds shared co-ancestry with sheep from China and Southwestern Asia (Iran).

**Conclusions:**

In this study, we derived the genetic characteristics of the major Russian local sheep breeds, which are moderately diverse and have a strong population structure. Pooling our data with a worldwide genotyping set gave deeper insight into the history and origin of the Russian sheep populations.

**Electronic supplementary material:**

The online version of this article (10.1186/s12711-018-0399-5) contains supplementary material, which is available to authorized users.

## Background

The sheep (*Ovis aries*) is one of the economically most important agricultural species and produces a wide range of valuable products including food (meat, milk) and raw materials (wool, sheepskin) [1]. Since their domestication approximately 11,000 years ago (YA) [2, 3], sheep have spread to all continents where they were reared under different environmental, management, and selection conditions. Consequently, diverse local breeds with a unique composition of various traits were developed.

Sheep breeding has always been an important branch of animal husbandry in Russia. The harsh climate conditions, which are characterized by low temperatures and 120 to 240 windy days per year, dictate a steady public demand for wool, sheepskins and felt products. Furthermore, Russia offers more than 75 million hectares of natural grasslands and pastures that are suitable for sheep rearing. Until 1990, Russia, along with Australia, China and New Zealand, was one of the world leaders in wool sheep production. However, the radical reformation of the economy reduced the number of sheep from 58 million in 1990, to 24.7 million in 2014 [4]. This trend was partly associated with a worldwide reduction of the demand of wool. Currently, sheep breeding is recovering and turning its production to meat instead of wool. Thus, the proportion of wool breeds has decreased from 90% in 1990 to 56% in 2014, while that of meat types has increased from 10 to 44% [5]. These developments threaten many wool breeds and they have even abolished several of them [6]. From the 45 breeds that were recorded in 1990, only 28 are still maintained [7]. Wool breeds comprise breeds with coarse wool and breeds with fine and semi-fine wool. The Russian coarse wool breeds originated from local sheep that were well adapted to the local environmental conditions of certain regions, such as the Edilbai and Kalmyk fat-rumped breeds in the hot dry steppe regions in the south of Russia, the Tuva short-fat-tailed breed in the Trans-Baikal area with a harsh continental climate, the Andean and Lezgin breeds in the mountain areas of the North Caucasus with poor forage resources, and Romanov sheep in the Central Russia with cold winters. The coarse wool breeds were created mainly by folk selection practices and were only slightly improved by crossbreeding with high-producing foreign breeds [8, 9]. Furthermore, the Russian coarse wool breeds exhibit a large diversity in tail fat deposition as well as in tail length, and they include the short-thin-tailed Romanov, the long-fat-tailed Kuchugur, Karakul and Caucasian Mountain breeds, the short-fat-tailed Buubei and Tuva, and the fat-rumped Edilbai and Kalmyk breeds.

The Russian semi-fine wool breeds were established from local ewes and were substantially influenced by the Romney and Lincoln breeds [10, 11]. Most of the Russian fine wool breeds were developed during the Soviet period by improving local breeds with low productivity, mainly through crossbreeding with Merino-derived breeds such as Rambouillet and Australian Merino sheep.

The development of high-throughput arrays for genotyping of multiple single nucleotide polymorphisms (SNPs) has revolutionized modern genetic studies [12, 13]. This technology allows unambiguous scoring and the combination of standardized data from different laboratories [14–16], thus providing a powerful tool to address a number of genetic issues [17, 18] including the successful application for studies on population structure in farm animals. During the last decade, detailed studies of the biodiversity and admixture levels in sheep breeds from Asia, Africa, America, Europe, Australia and New Zealand were performed using SNPs [19–23]. To date, only a few Russian sheep breeds have been genotyped using the OvineSNP50K BeadChip [24], whereas most of them have been analyzed using mitochondrial [25] and microsatellite markers exclusively [26–28].

In this work, we investigated the patterns of whole-genome diversity and the population structure of 25 local Russian sheep breeds using genome-wide genotype data. Furthermore, we determined the genetic relationship of the studied breeds with other breeds worldwide to elucidate the origin of the Russian sheep breeds.

## Methods

### Sample collection

**Fig. 1:**
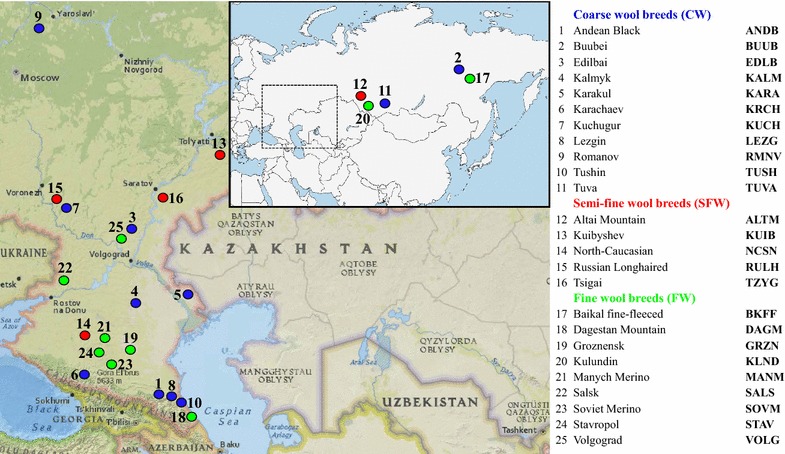
Map of sampling locations for this study. Both maps illustrate the geographical points where the samples of the 25 Russian sheep breeds were collected for this study. The coarse wool breeds are in blue; the semi-fine and fine wool breeds are in red and green colors, respectively. The numbers for the breeds are as follows: 1. Andean Black, 2. Buubei, 3. Edilbai, 4. Kalmyk, 5. Karakul, 6. Karachaev, 7. Kuchugur, 8. Lezgin, 9. Romanov, 10. Tushin, 11. Tuva, 12. Altai Mountain, 13. Kuibyshev, 14. North-Caucasian, 15. Russian Longhaired, 16. Tsigai, 17. Baikal fine-fleeced, 18. Dagestan Mountain, 19. Groznensk, 20. Kulundin, 21. Manych Merino, 22. Salsk, 23. Soviet Merino, 24. Stavropol, 25. Volgograd. For a description of the sheep breeds (see Additional file 1: Table S1, Additional file 2: Table S2)

Three hundred and ninety-six tissue samples were collected from 25 local Russian sheep breeds. These breeds included nine fine wool breeds (Baikal fine-fleeced, Dagestan Mountain, Groznensk, Kulundin, Manych Merino, Salsk, Soviet Merino, Stavropol, and Volgograd), five semi-fine wool breeds (Altai Mountain, Kuibyshev, North Caucasian, Russian Longhaired, and Tsigai), and 11 coarse wool breeds. The latter comprised the short-thin-tailed Romanov, the fat-tailed Andean Black, Buubei, Karakul, Karachaev, Kuchugur, Lezgin, Tushin, Tuva breeds, and the fat-rumped Edilbai and Kalmyk breeds (Table 1), (Fig. 1) and (see Additional file 1: Table S1). Tissue samples were collected by trained personnel under strict veterinary rules.

**Table 1:** Descriptive statistics of the genetic diversity of the 25 Russian sheep breeds analyzed

Breed	Code	N	H_E_	A_R_	*Ne*_5_	*Ne*_50_
*Coarse wool breeds*						
Andean Black	ANDB	16	0.358	1.900	109	594
Buubei	BUUB	17	0.367	1.916	103	629
Edilbai	EDLB	17	0.375	1.926	301	1572
Kalmyk	KALM	18	0.378	1.931	292	1626
Karachaev	KRCH	16	0.381	1.937	282	1678
Karakul	KARA	22	0.373	1.924	543	2171
Kuchugur	KUCH	16	0.353	1.898	65	357
Lezgin	LEZG	15	0.378	1.932	155	1195
Romanov	RMNV	26	0.354	1.890	216	620
Tushin	TUSH	9	0.378	1.931	85	709
Tuva	TUVA	16	0.377	1.929	234	1649
*Semi*-*fine wool breeds*						
Altai Mountain	ALTM	12	0.387	1.944	175	910
Kuibyshev	KUIB	15	0.390	1.946	272	955
North Caucasian	NCSN	16	0.383	1.936	242	708
Russian longhaired	RULH	16	0.370	1.918	186	487
Tsigai	TZYG	16	0.388	1.946	331	1547
*Fine wool breeds*						
Baikal fine-fleeced	BKFF	7	0.395	1.955	86	685
Dagestan Mountain	DAGM	16	0.384	1.939	318	942
Groznensk	GRZN	13	0.390	1.948	302	1542
Kulundin	KLND	16	0.373	1.920	123	552
Manych Merino	MANM	16	0.385	1.940	249	1031
Salsk	SALS	16	0.382	1.937	229	987
Soviet Merino	SOVM	14	0.387	1.944	302	1401
Stavropol	STAV	14	0.383	1.938	243	1005
Volgograd	VOLG	15	0.379	1.933	269	875

*CW* coarse wool breeds, *SFW* semi-fine wool breeds, *FW* fine wool breeds

H_E_ = unbiased expected heterozygosity, A_R_ = rarified allelic richness, *Ne*_5_ and *Ne*_50_ = effective population sizes back five and 50 generations, respectively

### DNA extraction and whole-genome SNP genotyping

Genomic DNA was extracted using Nexttec columns (Nexttec Biotechnology GmbH, Germany) following the manufacturer’s instructions. The concentrations of DNA solutions were determined using a NanoDrop-2000 (Thermo Fisher Scientific, Wilmington, DE, USA) and a Qubit 3.0 fluorimeter (Life Technologies). DNA concentrations and the OD260/OD280 ratio of DNA solutions were determined by NanoDrop. A Qubit dsDNA HS (high sensitivity, 0.2–100 ng) Assay Kit was used to measure the concentration of dsDNA according to the manufacturer’s protocols. The DNA quality was checked by 1% agarose gel electrophoresis. Whole-genome SNP genotyping was performed using the OvineSNP50 BeadChip (Illumina, San Diego, CA, USA).

### Construction of datasets

Two datasets were included in the analyses. The first one comprised 25 Russian sheep breeds (see Additional file 1: Table S1), while the second one included 24 of the 25 Russian sheep breeds mentioned above (except for the Baikal fine-fleeced breed, which was excluded from the combined dataset due to the small number of samples) and 2791 samples from 58 worldwide sheep breeds from publicly available sources [19, 21–23]. To account for the effects of family structures within the subpopulations, the genome-wide relationships between all animal pairs were inferred by estimating a unified additive relationship (UAR) matrix according to Yang et al. [29]. After exclusion of one of 1157 pairs of highly related animals (relationship > 0.25), the combined dataset comprised the SNP genotypes of 1592 relatively unrelated individuals from 82 breeds. Outliers were identified using a neighbor-joining tree based on identical-by-state (IBS) allele-sharing distances (–distance 1-ibs). Three outliers were found and removed from the Stavropol, Tushin, and Altai Mountain datasets.

The worldwide breeds were pooled according to their historical geographic origin and included 13 breeds from the British Isles, five breeds from Northern Europe, six breeds from Central Europe, 22 breeds from Southwestern Europe, three breeds from Asia, three breeds from Southwestern Asia, two breeds from South Africa, and four breeds from the Americas. Breed acronyms and color codes are available in Table S2 (see Additional file 2: Table S2).

### SNP quality control

First, the accuracy and efficiency of SNP genotyping were assessed. Valid genotypes for each SNP were determined by applying a cut-off of 0.5 for the GenCall (GC) and GenTrain (GT) scores [30]. Next, PLINK 1.07 [31] was used to exclude SNPs for which less than 90% of the individuals were genotyped (–geno 0.1), that had a minor allele frequency (MAF) lower than 5% (–maf 0.05), that departed from Hardy–Weinberg equilibrium at p < 10^−6^ (–hwe 1e-6) and that were in linkage disequilibrium (–indep-pairwise 50 5 0.5). Finally, only SNPs that are located on autosomes were kept for further analyses. Individuals with more than 10% missing genotypes (–mind 0.1) were removed. A Hardy–Weinberg equilibrium test was not performed for comparisons with worldwide breeds because too many SNPs would be excluded due to the Wahlund effect [32].

### Whole-genome SNP data processing

The R package ‘diveRsity’ [33] was used to calculate expected heterozygosity (H_E_) [34], rarefied allelic richness (A_R_) and pairwise *F*_ST_ values based on SNP genotypes. Multi-dimensional scaling (MDS) analysis based on pairwise identical-by-state (IBS) distances was performed with PLINK 1.07 (–cluster, –mds-plot 4) and visualized with the R package “ggplot2” [35]. Pairwise Nei’s genetic distances [36] were calculated using the R package ‘adegenet’ [37]. Neighbor-net graphs both for the Russian and the combined dataset based on pairwise *F*_ST_ values were computed using SplitsTree 4.14.5 [38].

Genetic admixture calculations were performed using Admixture v1.3 [39] and plotted with the R package “pophelper” [40]. Values of K (the number of assumed ancestral populations) ranging from 1 to 25 for the Russian dataset and from 1 to 74 for the combined dataset as well as their respective cross-validation (CV) errors were evaluated.

A map illustrating the area of sampling for each Russian sheep breed was obtained from the NatGeo Mapmaker Interactive database [41]. The outline map was plotted using the R package “maps” [42].

Trends of effective population size (*Ne*) were estimated from linkage disequilibrium (LD) as implemented in *SNeP* [43]. Default parameters were applied, except for the sample size correction, occurrence of mutation (α = 2.2; [44]), and recombination rate between a pair of genetic markers according to Sved and Feldman [45]. The most recent estimate of *Ne* was taken five generations back (*Ne*_5_). Furthermore, *Ne* estimates for *c *= 1 Mb (~ 50 generations ago; *Ne*_50_), where *c* is the distance between the SNPs in Morgans, were used for comparison with results from Kijas et al. [19, 23, 46]. A ‘*Ne* changing ratio’ (*NeC*) analysis was used as a proxy of the speed in *Ne* changes in the 20 most recent generations. The slope of each segment that links a pair of neighboring *Ne* estimates was calculated and normalized using the median of the most recent 20 *Ne* estimates.

R version 3.3.2 was used to create input files [47].

## Results

### Analysis of genetic diversity, population structure and genetic differentiation within 25 Russian sheep breeds

Descriptive statistics of the genetic diversity of the 25 Russian sheep breeds analyzed are in Table 1. Estimates of expected heterozygosity (H_E_) and rarified allelic richness (A_R_) in the Russian breeds under study were higher than 0.358 and 1.900, respectively. Only the Romanov breed had a lower level of genetic diversity with an H_E_ of 0.354 and A_R_ of 1.890.

The mean *Ne*_5_ value was around 228, with the Karakul and Kuchugur breeds displaying the highest (543) and lowest (65) values, respectively. The recorded *Ne*_*50*_ values showed a similar trend i.e. 2171 for the Karakul and 357 for the Kuchugur breeds.

**Fig. 2:**
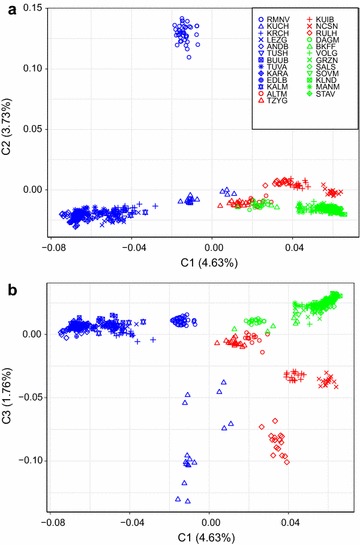
Multi-dimensional scaling (MDS) analysis of the Russian sheep breeds. The analysis was performed for the first two components (C1 and C2) (**a**) and for the first and third components (C1 and C3) (**b)**. The coarse wool breeds are indicated in blue; the semi-fine and fine wool breeds are in red and green colors, respectively. For a description of the sheep breeds (see Additional file 1: Table S1, Additional file 2: Table S2)

The first component of the MDS analysis **(**Fig. 2**)** accounted for 4.63% of the genetic diversity and discriminated Russian breeds with coarse wool from breeds with fine and semi-fine wool. The second component (3.73% of the genetic variability) clearly differentiated the Romanov breed from the remaining breeds. In general, the coarse wool and the fine wool breeds clustered into two distant groups with minor exclusions. According to the first and third components, the Kuchugur breed was positioned outside the cluster of coarse wool breeds (Fig. 2a). Regarding the fine wool breeds, the Dagestan Mountain and a few Baikal fine fleeced individuals were similar to the closely-related Tsigai and Altai Mountain breeds (Fig. 2a). The third component (Fig. 2b) provided a better understanding of the spatial distribution of the semi-fine wool breeds, which were separated from the other breeds, except for the Kuchugur breed. Furthermore, unlike the majority of the coarse and fine wool breeds, the semi-fine wool breeds did not form a united cluster.

**Fig. 3:**
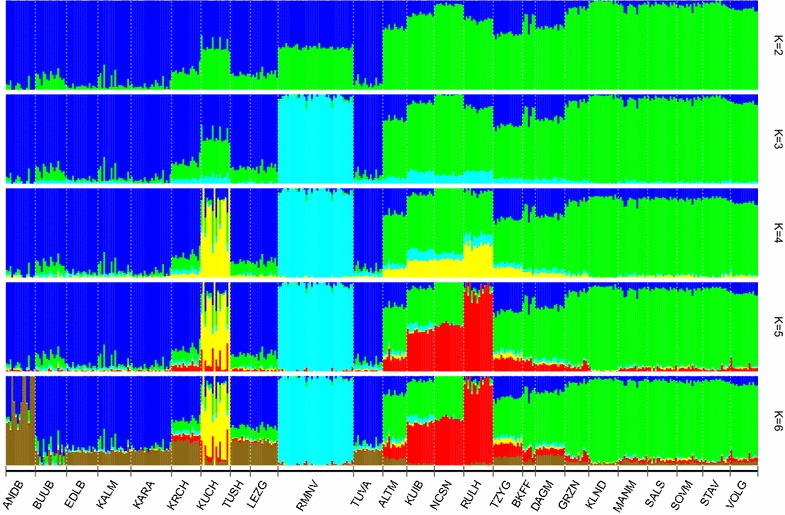
Cluster structure of the 25 Russian sheep breeds revealed by admixture analysis. For a description of the sheep breeds (see Additional file 1: Table S1, Additional file 2: Table S2)

The *F*_ST_ values computed for each pair of breeds (see Additional file 3: Table S3) and the pattern of the admixture analysis (Fig. 3) were in accordance with the MDS results. At K = 2, the Russian local breeds were separated into two main clusters according to wool type. The first cluster included the fine and semi-fine wool breeds and the second one comprised the coarse wool breeds. At K = 3, we found a strong genetic differentiation of the Romanov breed from all other studied breeds that persisted at higher K-values. The other breeds were distributed across the two remaining clusters according to wool type. The distinct genetic remoteness of the Romanov breed was consistent with the average pairwise *F*_ST_ values between the Romanov breed and the other breeds (*F*_ST_ = 0.084–0.124) and the MDS findings. At K = 4, a high degree of genetic heterogeneity was observed for the Kuchugur breed, which revealed mixed ancestry. Besides, from K = 4 to higher values, the group of semi-fine wool breeds (except for the Russian Longhaired breed) demonstrated admixed ancestry with a clear share of the genetic background from fine wool breeds. On the contrary, the Russian Longhaired breed was the most differentiated within the semi-fine wool cluster (*F*_ST_ = 0.046–0.059). A high genetic similarity was detected between the Altai Mountain and Tsigai breeds (*F*_ST_ = 0.013), and the North Caucasian and Kuibyshev breeds (*F*_ST_ = 0.020). The lowest cross-validation error was found at K = 6, at which slight changes were detected within the coarse wool cluster. Thus, an additional ancestral component was observed in the coarse wool breeds, which was most dominant in the native fat-tailed North Caucasian breeds (Andean Black, Karachaev, Lezgin and Tushin). The results of the analyses performed at higher K-values (K > 6) overlapped with the above-mentioned results.

**Fig. 4:**
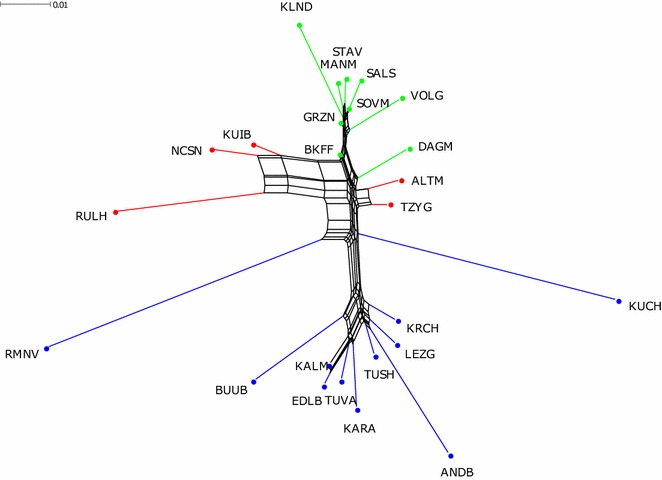
Neighbor-net graph of 25 Russian sheep breeds based on pairwise Fst values. The branches corresponding to the Russian coarse wool, semi-fine and fine wool breeds are indicated in blue, red and green, respectively. For a description of the sheep breeds (see Additional file 1: Table S1, Additional file 2: Table S2)

For the Russian breeds, the neighbor-net graph (Fig. 4) was in agreement with the MDS pattern. Thus, most of the fine wool and coarse wool breeds formed two distinct groups. The semi-fine wool breeds were positioned between the above-mentioned clusters. At the same time, the neighbor-net graph showed the subdivision within the wool types more precisely. Thus, within the cluster of fine-wool breeds, the Volgograd breed formed its own independent branch, while the Dagestan Mountain and Baikal fine-fleeced breeds were separated from the fine wool group. The short-thin-tailed Romanov and the fat-tailed Kuchugur breeds separated from the cluster of coarse wool breeds, which comprised an independent branch of the fat-tailed Buubei breed and two fat-tailed sub-clusters (Karachaev + Tushin + Lezgin + Andean Black and Edilbai + Kalmyk + Karakul + Tuva). The semi-fine wool breeds separated into two groups: Altai Mountain + Tsigai, and Russian longhaired + Kuibyshev + North Caucasian, which were positioned on the opposite edges of the graph.

**Fig. 5:**
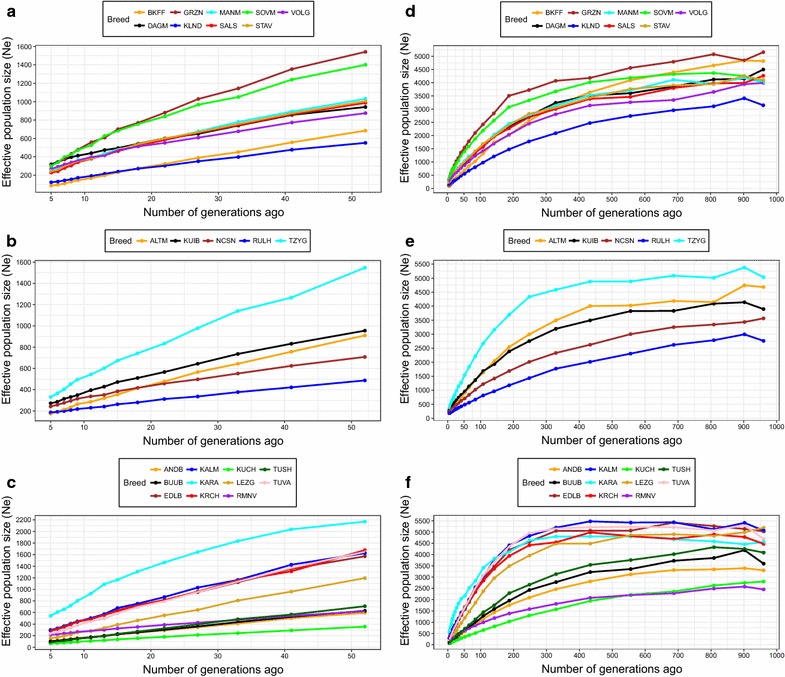
Historical effective population size (*Ne*) based on LD estimates. *Ne* values for 50–1000 generations ago are shown for the fine wool (**a**, **d**), semi-fine wool (**b**, **e**) and coarse wool (**c**, **f**) sheep breeds. For a description of the sheep breeds (see Additional file 1: Table S1, Additional file 2: Table S2)

The *NeC* analysis identified several sudden changes in effective population size (Fig. 5). Specifically, two major peaks of *Ne* decline were recorded around eight and 13 generations ago in 24 (excluded Dagestan Mountain) and 22 (excluded Karakul, Dagestan Mountain and Soviet Merino) breeds, respectively. Other strong signals of *NeC* were recorded around generations 6 (Stavropol, Soviet Merino, Salsk, Kuibyshev, Romanov) and 7 (Baikal fine-fleeced, Groznensk, Tsigai, Andean Black, Kuchugur, Kalmyk) (see Additional file 4: Figure S1).

### Phylogenetic relationships between Russian and global sheep breeds

**Fig. 6:**
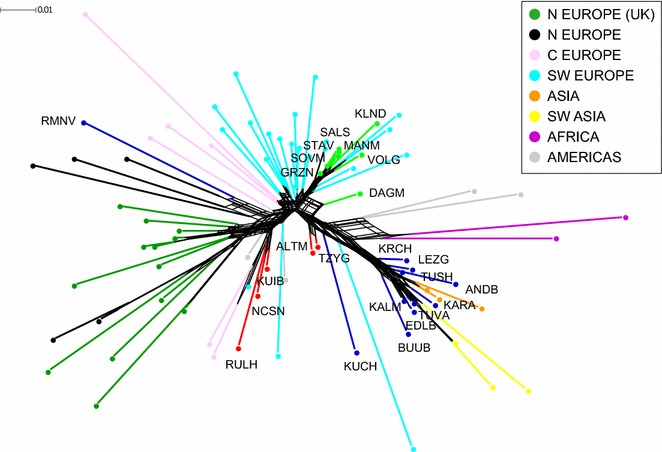
Neighbor-net graph of Russian and worldwide sheep breeds based on pairwise Fst values. The branches corresponding to the Russian coarse wool, semi-fine and fine wool breeds are indicated in blue, red and green, respectively. The colors of the branches of the worldwide breeds correspond to their ancestral geographic origin and are identical to the colors in Table S3 (see Additional file 3: Table S3): green for the British Isles, black for Northern Europe, pale pink for Central Europe, cyan for Southwestern Europe, orange for Asia, yellow for Southwestern Asia, purple for Africa and gray for the Americas. For a description of the sheep breeds (see Additional file 1: Table S1, Additional file 2: Table S2)

To study the ancestry of the Russian sheep breeds, we pooled our data with publicly available genotype data of 58 sheep breeds from across the world [19, 21–23]. The neighbor-net analysis, (Fig. 6) showed a clear pattern of consistent subdivision among the wool types as also evidenced by MDS (Fig. 2) and admixture results (Fig. 3). Accordingly, the fine wool breeds were clustered among the Merino and Merino-derived sheep breeds except for the Dagestan Mountain breed, which branched individually between the fine-wool cluster and the coarse-wool and crossbreed sheep breeds of the Americas and Africa. The fat-tailed Russian coarse wool breeds were clustered into one group with the Asian and Southwestern Asian sheep. The Romanov breed showed a clear Northern European origin and clustered together with the Finnsheep and Norway Spaelsau breeds. The Kuchugur breed was separated from the cluster of coarse wool sheep breeds. The semi-fine wool breeds split into two groups, with one including the Altai Mountain and Tsigai breeds and the other comprising the Kuibyshev, Russian Longhaired and North Caucasian breeds that clustered with Swiss, European and American crossbreeds and adjoined a group of sheep from the British Isles.

**Fig. 7:**
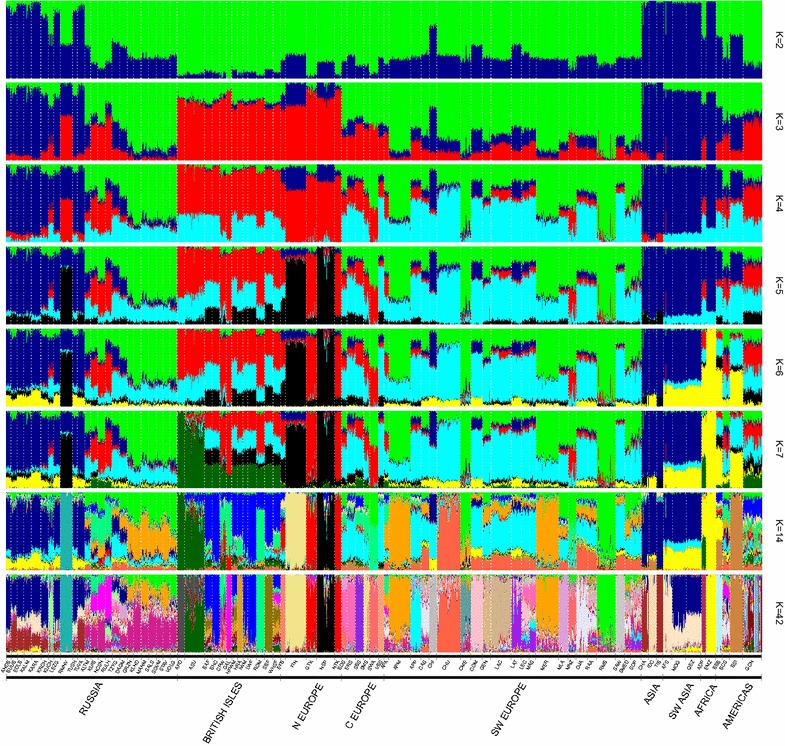
Bar plot showing the extent of admixture of the Russian sheep breeds from worldwide breeds. Breed codes are indicated at the bottom of the bar plots. The breeds are grouped according to their ancestral geographic origin (Russia, the British Isles, Northern Europe, Central Europe, Southwestern Europe, Asia, Southwestern Asia, Africa and the Americas) and arranged in the order indicated in Table S2 (see Additional file 2: Table S2). For a description of the Russian sheep breeds (see Additional file 1: Table S1, Additional file 2: Table S2) and of the worldwide sheep breeds, (see Additional file 2: Table S2)

The results of the model-based admixture clustering (Fig. 7) were consistent with those of the neighbor-net analysis. At K = 2, we observed that most of the local Russian fat-tailed coarse wool sheep breeds showed high similarity with Asian breeds (blue color), whereas for the Romanov and Kuchugur breeds this trend did not predominate. At K = 3, we detected a differentiated cluster including sheep from the British Isles and Northern Europe. It was obvious that their genetic background was shared with that of the Romanov and semi-fine wool Russian breeds as well as sheep from both Americas. At K = 4, the genetic background of the Merino breeds (Merino, Rambouillet, Australian Poll Merino) was clearly present in the Merino-derived fine wool Russian breeds. At K-values from 5 to 7, the Romanov breed showed high genetic relatedness to the other Northern short-tailed breeds (Finnsheep and Norway Spaelsau), but a K value of 14 clearly differentiated the Romanov breed. According to the cross-validation error, the largest number of founder populations was 42. The fine wool Russian breeds with Merino and Rambouillet genetic backgrounds formed their own genetic group with a complex ancestry. The semi-fine wool breeds were close to the cluster of fine wool breeds but were obviously admixed with sheep breeds of the British Isles. We identified a relatively large Romney Marsh ancestry in the Kuibyshev and the North Caucasian breeds, while the Russian Longhaired breed showed a strong Galway component (such as the long-wool Lincoln breed) and admixture with the Kuchugur breed.

The global admixture analysis revealed that the genetic backgrounds that predominate in Chinese and Iranian sheep are present in all Russian coarse wool breeds except for the Romanov and Kuchugur breeds. In addition, the fat-rumped Edilbai and Kalmyk as well as the short-fat-tailed Buubei and Tuva breeds shared a significant common genetic ancestry with Chinese (Tibet) sheep. We detected similar patterns for the Russian Karakul and the Iran Afshari breeds. Most of the Russian sheep breeds analyzed here revealed a complex ancestry, but two Russian indigenous breeds (Romanov and Kuchugur) formed specific genetic patterns that were not detected in the other studied sheep populations. We observed a high level of consolidation for the Romanov breed, while the extent of admixture for the Kuchugur breed was more obvious.

## Discussion

Due to their vast extension and unique Eurasian geographical position, Russian local livestock are of special interest [26, 48–50]. The first key point of interest for us was to investigate the whole-genome diversity of the breeds under study. This was crucial since no Russian sheep breeds were included in the OvineSNP50 BeadChip (Illumina) discovery panel. We found that the levels of variability of Russian breeds were similar to those reported for other sheep breeds [19, 21–23].

Regarding the slope changes in the *Ne* trend lines (see Additional file 4: Figure S1), the major peak of *Ne* decline for 24 of the 25 breeds analysed occurred about eight generations ago. This decline is most likely due to the beginning of the restructuring of the Soviet economy, the so-called Perestroika, which resulted in the destruction of the planned economy system and in a deep crisis of the agricultural sector. The subsequent lack of forage and food resources led to a considerable decrease in the number of all livestock populations including sheep, which can be detected in the evolution of the *Ne*. The negative consequences continued during the next decade of the post-soviet times, which could explain the shifts of the peaks in the *Ne* slopes of some breeds between 6 and 8 generations ago. However, one breed i.e. the Dagestan Mountain breed did not follow this trend and maintained its population size during the Perestroika. A possible explanation for this trend might be the great popularity of the Dagestan Mountain sheep in their breeding region because of their combined good meat and wool productivity. In addition, we observed that the coarse wool breeds did not display any further recent significant peaks, whereas fine and semi-fine wool breeds do. This could be indirectly associated with the growing interest of farmers in local coarse wool breeds that are highly adapted to specific regions.

We observed a decline in *Ne* over time for the breeds analyzed (Fig. 5). The most rapid decline in *Ne* occurred over the last 200 to 400 generations in all breeds. In general, this decrease corresponded to the results obtained by Kijas et al. [19] on sheep breeds included in the HapMap Project data [51]. However, some breeds showed interesting patterns regarding changes in ancestral *Ne*. Until 250 generations ago, the *Ne* curve of the Tsigai breed was almost parallel to the x-axis. The same tendency towards smooth curves until 200 to 250 generations ago was also observed for the Tuva, Karachaev, Kalmyk, Edilbai, Karakul and Lezgin breeds. This pattern most likely reflects their ancient origin and wide geographic distribution. In addition, all mentioned breeds currently have large *Ne* (Table 1). However, in their latest study, Prieur et al. [52] suggested that the 50K SNP BeadChip is not suitable for estimating the *Ne* more than 100 generations ago. Consequently, these inferences onto many generations ago based on a 50K DNA array data should be treated with caution.

Overall, the current effective population size estimates (*Ne*_50_) for the Russian sheep groups were larger than those of the other worldwide sheep breeds [19, 23, 46]. The Kuchugur breed recorded the smallest *Ne*_5_ and *Ne*_50_ values (65 and 357, respectively), which most likely reflect the low management conditions of the breed, for which no precise information on the population size is available [53]. However, although the *Ne*_50_ values are not as critical as those for Dorset Horn (*Ne*_50_ = 134) and Wiltshire (*Ne*_50_ = 100) breeds [19], the most recent *Ne*_5_ estimate for the Kuchugur breed is around 50, which is considered as the threshold risk of extinction in the short term [54]. This implies that the breed should be monitored closely as a relevant candidate for conservation efforts.

### On the history of the Russian coarse wool sheep breeds

The analysis of a combined dataset of local and worldwide sheep genotypes allowed us to gain insight into the history and ancestry of the Russian sheep population. The Russian coarse wool breeds are characterized by differences in tail phenotypes and included sheep with thin tails and sheep with fat tails and fat rumps. Among these different tail types, the thin tail is likely to be the ancestral trait, since it is present in the mouflon, which is the most probable wild ancestor of modern sheep. According to archaeological findings, fat-tailed sheep were developed from thin-tailed sheep and were first mentioned about 5000 years ago [55]. In this regard, fat deposition in the tail is an important genetic trait that is considered one of the major post-domestic adaptations to harsh environments (drought seasons, extreme cold winters and food shortages) as well as an energy source for long migrations [56, 57]. In our study, the tail types of the Russian coarse wool breeds could provide valuable information on their origin.

Here, we recorded a strong differentiation between the thin-tailed Romanov and the local fat-tailed and fat-rumped groups (Figs. 2, 3, 4, 6, and 7). A further subdivision was detected within the group with fat deposition in the tail. This group comprised the long-fat-tailed Kuchugur breed and two subclusters: Karakul (long-fat-tailed), Buubei and Tuva + Edilbai + Kalmyk (short-fat-tailed and fat-rumped), and Andean Black + Lezgin + Tushin + Karachaev (long-fat-tailed). For a better understanding of the results, some aspects of the origin of each breed are discussed below.

The Romanov breed, which is the only short-thin-tailed Russian coarse wool breed, was created by local farmers in the seventeenth century in the Yaroslavl region. Today, the Romanov breed is famous worldwide for its extraordinary prolificacy, early sexual maturity and out-of-season breeding ability [8]. Compared with the other coarse wool breeds, the Romanov breed clearly showed different ancestry, which was well demonstrated by the results at the local level (Figs. 2, 3, and 4). Neighbor-net (Fig. 6) and admixture graphs (Fig. 7) confirmed the North European genetic roots of the breed. Indeed, the Romanov breed clustered outside the other Russian coarse wool breeds and formed a group with the Finnsheep and Norwegian Spaelsau breeds (Fig. 6). Romanov and Finnsheep are the most well known and numerous representatives of the Northern European short-tailed breeds [49, 58]. It is believed that Norse Vikings spread these northern sheep to several countries from the late eighth century to the middle of the eleventh century AD [59]. The patterns obtained at K = 5, 6 and 7 (Fig. 7) also suggested a common ancestry between Romanov and Finnsheep. However, at K = 14 and higher, all breeds clearly differentiated from one another (Fig. 7). Originating from the same ancient Nordic ancestor group, each breed (including Romanov) most likely formed their unique gene pool under different selection, geographical and feed conditions. Such interpretation is in agreement with historical records, which consider the Romanov an independent branch of the Northern European short-tailed breeds [60].

Neighbor-net and admixture graphs (Figs. 6, 7) suggested a common ancestry between the fat-tailed Russian coarse wool breeds, Asian (Chinese and Indian), and Southwestern Asian (Iran) sheep. The range of the fat-tailed and fat-rumped sheep overlaps with the European and Asian Russian territory, which was proposed to be the consequence of nomadic expansions including invasions and the intensive east–west trading via the Silk Road [57, 61, 62]. Specifically, sheep from the Middle Eastern domestication center were brought to the Caucasus, the area east of the Caspian Sea and Central Asia, and finally arrived in North and Southwest China and the Indian subcontinent via the Mongolian Plateau region [57, 62]. Furthermore, the gene flow could have taken place through the major Turkic migrations and later Mongol invasions [57, 61], which were accompanied by sheep flocks. Indeed, this may explain the admixture of Caucasian Mountain fat-tailed sheep and the Chinese breeds.

The fat-tailed local sheep, Andean, Karachaev, Lezgin, and Tushin formed the Caucasian Mountain fat-tailed cluster. Sheep husbandry has always been of special value to the Russian south regions, especially in mountain regions, and it represents an inseparable part of the local cultural heritage. Andean, Karachaev, Lezgin, and Tushin sheep are versatile breeds that produce meat, wool and milk in equivalent proportions. These sheep easily withstand long marches over great distances and are highly adapted to grazing the mountain and lowland pastures. The wool is used for manufacturing felt shoes and fabrics to sew the traditional men’s clothing. All these breeds were created by folk selection practices during the nineteenth and twentieth century in different mountain parts of the North Caucasus [63, 64].

The second cluster of the fat-tailed local sheep included breeds with more significant Asian ancestry (China and Tibet): Kalmyk, Edilbai, Buubei and Tuva. The fat-rumped Edilbai and Kalmyk sheep combine high meat and grease productivity with excellent adaptability to year-round grazing in extreme semi-desert and desert climatic conditions [6]. Although the breeds are reared mostly in the southern part of Russia (Fig. 1) and (see Additional file 1: Table S1), they are of Asian ancestry. Thus, the Edilbai breed was obtained by crossing Astrakhan rams with Kazakh fat-rumped ewes between the Ural River and the Volga River. The Kalmyk originated from indigenous fat-rumped sheep from China and improved with sheep from the Edilbai and Torgudsk breeds. The close relation between Edilbai and Kalmyk sheep was very well illustrated by the formation of a common branch in the neighbor-net (Fig. 4) and by the low pairwise *F*_ST_ value (*F*_ST_ = 0.007), (see Additional file 3^3^ Table S3).

The Buubei breed is the result of long-term improvement of the indigenous Buryat sheep. This breed is characterized by a high prolificacy and good adaptation to the severe climatic conditions of the Republic of Buryatia [65, 66]. In the middle of the twentieth century, the indigenous Buryat sheep had become extinct [65]. In the 1980s’, a small group of indigenous Buryat sheep was found in China and was later transported to their historic homeland. This is compatible with our findings that the Chinese genetic background significantly contributed to the Buubei breed.

The ancient Tuva breed was raised under the harsh climate of the Republic of Tyva by local nomadic tribes approximately 2000 YA. These sheep can survive on small amounts of forage while accumulating body fat and they can take snow instead of water, which is an important advantage for surviving in steppe and mountain pastures. Their coarse wool, which is composed of down, guard and dead hair, is the feedstock for shoes and felt fabrics for traditional clothing [67]. The Republic of Tyva has a common border with Mongolia across which the gene flow with China could have taken place. Furthermore, both Buubei and Tuva are short fat-tailed and are very similar to Chinese breeds. A study of the demographic history of Chinese native sheep showed that the expansion of short-fat-tailed sheep into China was mainly associated with the invasions of Mongols, who reared the short-fat-tailed sheep, from the Mongolian Plateau during the twelvetieth and thirtieth centuries [62]. Consequently, the Buubei, Tuva and Chinese breeds probably share Mongolian ancestry.

The position within the fat-tailed coarse wool group of the Russian Karakul breed is not perfectly clear. The local neighbor-net (Fig. 4) suggested a closer relation with the Kalmyk, Edilbai and Tuva breeds. However, the global admixture results (Fig. 7) showed significant co-ancestry between the Karakul and Iranian breeds, which is more consistent with the breed’s origin. The history of the creation of the Karakul breed is still in question and there are two main theories. Some scientists believe that the Karakul breed results from crossing the black indigenous sheep of Bukhara (Turkestan) with Afghan and native fat-rumped sheep [68]. Others assumed that the Arabs brought the ancestors of the Karakul breed to Middle Asia in the eighth century [69]. Both theories agree with our findings.

The long-fat-tailed Kuchugur showed a pattern of admixture that was quite similar to that of the other fat-tailed Russian coarse wool breeds at K = 5, 6, 7 and 14 (Fig. 7). However, Kuchugur appeared as an outlier according to the neighbor-net analyses (Figs. 4 and 6), with a branch that is positioned between the Tsigai + Altai Mountain cluster (with lower genetic distance) and the fat-tailed local cluster. This most likely reflects the crossbred origin of the Kuchugur breed. It is assumed that the Kuchugur breed resulted from the cross of indigenous crossbred coarse wool ewes with large Voloshian (Valakhian) rams [70]. Furthermore, the lowest pairwise *F*_ST_ value for the Kuchugur breed was detected with the Tsigai breed (*F*_ST_ = 0.068) (see Additional file 3: Table S3). Since both the Tsigai and Voloshian breeds originated in the Balkans, they are genetically close and have influenced many sheep breeds in Eastern Europe [71–74], which also confirms the European ancestry of Kuchugur. Moreover, historical records suggest that a foreign breed—most likely one of the English Longwool type—was used to improve the local crossbreds towards curly wool and good body conformation [75].

### On the history of the Russian semi-fine wool sheep breeds

Analysis of the phylogeny of the Russian semi-fine wool breeds revealed several ancestry backgrounds. The local neighbor-net analysis indicated the presence of two main clusters of which one includes the Altai Mountain and Tsigai breeds and the other the Kuibyshev, North Caucasian and Russian Longhaired breeds. The history of the creation of these breeds’ provided insight into this differentiation.

Both admixture patterns (Figs. 3, 7) showed a common genetic background for the Tsigai and Altai Mountain breeds. The Roman origin of the Tsigai sheep and its subsequent spread in the Balkans was previously suggested [73, 74, 76]. The history of the Russian Tsigai began when Transylvanian farmers brought Tsigai sheep from Romania to the former Russian Empire in 1914 [75–77]. Since the establishment of the Tsigai herd book, this breed was kept pure. However, possible admixture with fine wool breeds could probably have taken place at the early stages of Tsigai breeding after the breed was imported to Russia. Unfortunately, no original Romanian Tsigai SNP data is available to better evaluate the relationship between Russian and Romanian Tsigai sheep.

The Altai Mountain breed resulted from crossing local coarse wool sheep with the Groznensk breed, as confirmed by the admixture analysis (Figs. 3, 7). Furthermore, the Tsigai breed was involved in the breeding process of the Altai Mountain breed during the period from 1945 to 1970 [53, 70]. Their common ancestry is illustrated by the MDS, admixture plots and neighbor-net analyses (Figs. 2, 3 and 4), and confirmed by the low pairwise *F*_ST_ values (*F*_ST_ = 0.013) (see Additional file 3: Table S3).

The origin of the other semi-fine wool sheep was closely associated with the English long-wool breeds. Thus, the Kuibyshev breed was obtained from an ancestry that involved Romney Marsh rams [78]. At the first stages of the North Caucasian breed creation, both Romney Marsh and Lincoln rams were widely used. Because the Lincoln progeny showed higher growth rates and were characterized by a better external phenotype, only Lincoln rams were maintained in the breeding process [10, 11, 53]. Nevertheless, due to the close genetic relatedness between North Caucasian and Kuibyshev sheep (*F*_ST_ = 0.020), we assume that the Romney Marsh genetic background is still present in the modern North Caucasian sheep. The shared ancestry of both breeds and Romney Marsh was identified by the admixture analysis (Fig. 7). Interestingly, the neighbor-net analysis identified some genetic overlap between the North Caucasian and the Russian longhaired breeds (Fig. 6), which is consistent with the origin of the Russian Longhaired breed that was created with the participation of Lincoln sheep (see Additional file 1: Table S1), and by a relatively large Galway ancestry component, the Galway breed being a long-wool breed as the Lincoln breed (Fig. 7). Finally, Kuchugur is believed to have been involved in the development of the Russian Longhaired breed [10]. Although *F*_ST_ values between these breeds were significant (*F*_ST_ = 0.09), the presence of the Kuchugur background was obvious in the Russian Longhaired at K = 42 in the global admixture plot (Fig. 7).

### On the history of the Russian fine wool sheep breeds

Ciani et al. [22] conducted a study that focused on the Merino influence on the development of new breeds distributed throughout the world; however, the Russian Merino-derived sheep breeds were not included in the analysis. In the former USSR, wool production was one of the most prioritized branches of animal husbandry. In this regard, the majority of Russian fine wool breeds were created between 1920 and 1980. Thus, most fine wool breeds (Groznensk, Stavropol, Soviet Merino and Salsk) result from the improvement of local fine wool Mazaev and Novocaucasian ewes with commercial rams that have a high wool productivity such as the Spanish Merino, French and American Rambouillet, and Merino Précoce breeds [22, 70, 79].

The Manych Merino breed was developed from Stavropol ewes that were improved with Australian Merino rams [53]. The close genetic relationship between Manych Merino and Stavropol was evidenced by both by the neighbor-net analyses (Figs. 4 and 6), and by their low *F*_ST_ value (0.012) (see Additional file 3: Table S3). The Volgograd sheep resulted from a complex crossing that involved Groznensk rams [53] as suggested by the results of the neighbor-net analysis (Fig. 4) and the *F*_ST_ value (0.018) (see Additional file 3: Table S3).

Later, from 1990 to 2004, Australian Merino sheep were used to improve the quality of the wool of most of the Russian fine wool breeds [80]. However, the genetic background of the Dagestan Mountain and Baikal fine-fleeced breeds is clearly different to that of other local fine wool breeds (Fig. 2). This could most likely be due to the fact that local crossbred coarse wool ewes, specifically Gunib for Dagestan Mountain sheep and Buryat-Mongolian for Baikal fine-fleeced sheep, were used instead of Mazaev and Novocaucasian Merino sheep [81]. Nonetheless, an authentic Russian origin of the fine- and semi-fine-wool sheep is indicated by the K = 42 pattern of the global admixture plot (Fig. 7), in which these breeds share a (violet) ancestral component that is not present in any other breed.

## Conclusions

In this study, we investigated the genome-wide diversity and population structure of 25 Russian local sheep breeds for the first time. We identified three clusters corresponding to the wool type. We identified a main discriminating factor within the Russian coarse wool cluster i.e. tail type, with the short-thin-tailed Romanov breed clearly differentiated from the other fat-tailed or fat-rumped breeds. The combination of local Russian sheep data with a worldwide sheep SNP genotyping set provided admixture patterns that gave deeper insights into the origin of the local Russian sheep. Thus, our findings suggest shared ancestry of local fat-tailed coarse wool breeds and Southwestern Asian (Iran) sheep, which may be a consequence of nomadic migrations, including invasions and east–west trading. Although co-ancestry between the Romanov breed and the Northern short-tailed group was clearly confirmed, we also noted that this breed is genetically distinct, which may be clarified by future studies using a larger sample size, denser SNP panels or whole-genome sequencing. The computation of the most recent effective population sizes revealed a few local breeds with critically small values that constitute a warning flag for the implementation of conservation efforts (e.g. the Kuchugur breed). This study is the first step to design a more effective selection and conservation program for Russian local sheep breeds based on whole-genome SNP genotyping data. This is essential for sustainable sheep breeding at the global level and for the future prosperity of sheep breeding at the local level across Russia.

## Additional files

**Additional file 1: Table S1** Short description of the Russian sheep breeds under study.

**Additional file 2: Table S2** The joint dataset used in the study. Description: This table provides information on the joint dataset, which includes the Russian sheep breeds and sheep breeds from across the world. The breeds are grouped according to their ancestral geographic origin (Russia, the British Isles, Northern Europe, Central Europe, Southwestern Europe, Asia, Southwestern Asia, Africa and the Americas). The table presents the information concerning the breeds’ abbreviation and color representation for the geographical group of breeds, the sample size, country (region) of sample collection, and the references where the genotyping data were previously published.

**Additional file 3: Table S3** Genetic differentiation of 25 Russian sheep breeds based on Weir and Cockerham’s fixation index (*F*_ST_). This table provides information about *F*_ST_ values between the Russian breeds under study. The breeds’ groups of the same wool type are framed in blue (for coarse wool breeds), red (for semi-fine wool breeds) and green (for fine wool breeds). For a description of the sheep breeds (see Additional file 1: Table S1, Additional file 2: Table S2).

**Additional file 4: Figure S1** Slope changes in historical effective population size (*Ne*) trends. The graphs show the changes in slope trends for historical effective population size (*Ne*) for the period starting from approximately 18 generations ago for the Russian sheep breeds with the coarse wool (above), semi-fine wool (in the middle) and fine wool (below). For a description of the sheep breeds (see Additional file 1: Table S1, Additional file 2: Table S2).

## References

[1] Chessa B, Pereira F, Arnaud F, Amorim A, Goyache F, Mainland I (2009). Revealing the history of sheep domestication using retrovirus integrations. Science.

[2] Zeder MA (2008). Domestication and early agriculture in the Mediterranean Basin: origins, diffusion, and impact. Proc Natl Acad Sci USA.

[3] Vigne JD, Carrère I, Briois F, Guilaine J (2011). The early process of mammal domestication in the near east: new evidence from the pre-neolithic and pre-pottery neolithic in Cyprus. Curr Anthropol.

[4] IWTO Market Information, FAOSTAT. http://www.fao.org/faostat/en/#home. Accessed 15 Sept 2016.

[5] Lescheva M, Ivolga A (2015). Current state and perspectives of sheep breeding development in Russian modern economic conditions. Econ Agric.

[6] Erokhin AI (2014). Ovtzevodstvo.

[7] Amerkhanov KhA. Ovtzevodstvo I kozovodstvo Rossiiskoy Federatsii v tsyfrakh. Stavropol: BI; 2015 (**in Russian**).

[8] Veniaminov AA (1984). Porody ovets mira.

[9] Zakharov IA (2006). Genefondy sel`skokhozyastvennych zhivotnykh: geneticheskie resursy zhivotnovodsrva Rossii.

[10] Sel’kin II, Sokolov AN (2002). Sozdanie i soversenstvovanie polytonkorunnykh porod ovets. Ovtsy, kosy, sherstyanoe delo.

[11] Sel’kin II, Aboneev VV. Severokavkazskay myaso-sherstnaya poroda. Stavropol: BI; 2007 (**in Russian**).

[12] LaFramboise T (2009). Single nucleotide polymorphism arrays: a decade of biological, computational and technological advances. Nucleic Acids Res.

[13] Lenstra JA, Groeneveld LF, Edin GH, Kantanen J, Williams JL, Taberlet P (2012). Molecular tools and analytical approaches for the characterization of farm animal genetic diversity. Anim Genet.

[14] Morin PA, McCarthy M (2007). Highly accurate SNP genotyping from historical and low-quality samples. Mol Ecol Resour.

[15] Smith M, Pascal C, Grauvogel Z, Habicht C, Seeb J, Seeb L (2011). Multiplex preamplification PCR and microsatellite validation allows accurate single nucleotide polymorphism (SNP) genotyping of historical fish scales. Mol Ecol Resour.

[16] Kawęcka A, Gurgul A, Miksza-Cybulska A (2016). The use of SNP microarrays for biodiversity studies of sheep: a review. Ann Anim Sci.

[17] Gill P (2001). An assessment of the utility of single nucleotide polymorphisms (SNPs) for forensic purposes. Int J Legal Med.

[18] Paschou P, Ziv E, Burchard EG, Choudhry S, Rodriguez-Cintron W, Mahoney MW (2007). PCA-correlated SNPs for structure identification in worldwide human populations. PLoS Genet.

[19] Kijas JW, Lenstra JA, Hayes B, Boitard S, Porto Neto LR, San Cristobal M (2012). Genome-wide analysis of the world’s sheep breeds reveals high levels of historic mixture and strong recent selection. PLoS Biol.

[20] Zhang L, Mousel MR, Wu X, Michal JJ, Zhou X, Ding B (2013). Genome-wide genetic diversity and differentially selected regions among Suffolk, Rambouillet, Columbia, Polypay, and Targhee sheep. PLoS One.

[21] Ciani E, Crepaldi P, Nicoloso L, Lasagna E, Sarti FM, Moioli B (2014). Genome-wide analysis of Italian sheep diversity reveals a strong geographic pattern and cryptic relationships between breeds. Anim Genet.

[22] Ciani E, Lasagna E, D’Andrea M, Alloggio I, Marroni F, Ceccobelli S (2015). Merino and Merino-derived sheep breeds: a genome-wide intercontinental study. Genet Sel Evol.

[23] Beynon SE, Slavov GT, Farré M, Sunduimijid B, Waddams K, Davies B (2015). Population structure and history of the Welsh sheep breeds determined by whole genome genotyping. BMC Genet.

[24] Deniskova TE, Dotsev AV, Wimmers K, Reyer H, Kharzinova VR, Gladyr EA (2016). Genomic evaluation and population structure of eleven Russian sheep breeds. J Anim Sci.

[25] Tapio M, Marzanov N, Ozerov M, Cinkulov M, Gonzarenko G, Kiselyova T (2006). Sheep mitochondrial DNA variation in European, Caucasian, and Central Asian areas. Mol Biol Evol.

[26] Tapio M, Ozerov M, Tapio I, Toro MA, Marzanov N, Cinkulov M (2010). Microsatellite-based genetic diversity and population structure of domestic sheep in northern Eurasia. BMC Genet.

[27] Zinovieva NA, Selionova MI, Gladyr EA, Petrovic MP, Caro Petrovic V, Ruzic MD (2015). Investigation of gene pool and genealogical links between sheep breeds of southern Russia by blood groups and DNA microsatellites. Genetika.

[28] Deniskova TE, Selionova MI, Dotsev AV, Bobryshova GT, Gladyr EA, Kostjunina OV (2016). Variability of microsatellites in sheep breeds raced in Russia. Agric Biol [Sel`skokhozyastvennaya biologia].

[29] Yang J, Benyamin B, McEvoy BP, Gordon S, Henders AK, Nyholt DR (2010). Common SNPs explain a large proportion of the heritability for human. Nat Genet.

[30] Fan JB, Oliphant A, Shen R, Kermani BG, Garcia F, Gunderson KL (2003). Highly parallel SNP genotyping. Cold Spring Harb Symp Quant Biol.

[31] Purcell S, Neale B, Todd-Brown K, Thomas L, Ferreira MAR, Bender D (2007). PLINK: a tool set for whole-genome association and population-based linkage analyses. Am J Hum Genet.

[32] Wahlund S (1928). Zusammensetzung von Populationen und Korrelationerscheinungen vom Standpunkt der Vererbungslehre aus betrachtet. Hereditas.

[33] Keenan K, McGinnity P, Cross TF, Crozier WW, Prodohl PA (2013). diveRsity: an R package for the estimation of population genetics parameters and their associated errors. Methods Ecol Evol.

[34] Nei M (1978). Estimation of average heterozygosity and genetic distance from small number of individuals. Genetics.

[35] Wickham H (2009). ggplot2: elegant graphics for data analysis.

[36] Nei M (1972). Genetic distance between populations. Am Nat.

[37] Jombart T (2011). Ahmed I. adegenet 1.3-1: new tools for the analysis of genome-wide SNP data. Bioinformatics.

[38] Huson DH, Bryant D (2006). Application of phylogenetic networks in evolutionary studies. Mol Biol Evol.

[39] Alexander DH, Novembre J, Lange K (2009). Fast model-based estimation of ancestry in unrelated individuals. Genome Res.

[40] Francis RM (2017). POPHELPER: an R package and web app to analyse and visualise population structure. Mol Ecol Resour.

[41] NatGeo Mapmaker Interactive database. https://mapmaker.nationalgeographic.org/. Accessed 15 Dec 2017.

[42] maps: Draw Geographical Maps. https://CRAN.R-project.org/package=maps. Accessed 15 Dec 2017.

[43] Barbato M, Orozco-terWengel P, Tapio M, Bruford MW (2015). SNeP: a tool to estimate trends in recent effective population size trajectories using genome-wide SNP data. Front Genet.

[44] Corbin LJ, Liu AY, Bishop SC, Woolliams JA (2012). Estimation of historical effective population size using linkage disequilibria with marker data. J Anim Breed Genet.

[45] Sved J, Feldman M (1973). Correlation and probability methods for one and two loci. Theor Popul Biol.

[46] Barbato M, Hailer F, Orozco-terWengel P, Kijas JW, Mereu P, Cabras P (2017). Genomic signatures of adaptive introgression from European mouflon into domestic sheep. Sci Rep.

[47] R Core Team. R: a language and environment for statistical computing. R Foundation for statistical computing. Vienna, Austria; 2012. http://www.R-project.org.

[48] Tapio I, Tapio M, Grislis Z, Holm LE, Jeppsson S, Kantanen J (2005). Unfolding of population structure in Baltic sheep breeds using microsatellite analysis. Heredity (Edinb).

[49] Tapio M. Origin and maintenance of genetic diversity in North European sheep. PhD thesis, University of Oulu; 2006.

[50] Tapio M, Tapio I, Grislis Z, Holm LE, Jeppsson S, Kantanen J (2005). Native breeds demonstrate high contributions to the molecular variation in northern European sheep. Mol Ecol.

[51] International Sheep Genomics Consortium. http://www.sheephapmap.org/pag.php. Accessed 20 August 2017.

[52] Prieur V, Clarke SM, Brito LF, McEwan JC, Lee MA, Brauning R (2017). Estimation of linkage disequilibrium and effective population size in New Zealand sheep using three different methods to create genetic maps. BMC Genet.

[53] Dunin IM, Dankvert AG (2013). Spravochnik porod i tipov sel`skokhozyastvennykh zhivotnykh, razvodimykh v Rossiiskoi Federatsii.

[54] Taberlet P, Valentini A, Rezaei HR, Naderi S, Pompanon F, Negrini R (2008). Are cattle, sheep, and goats endangered species?. Mol Ecol.

[55] Ryder ML (1983). Sheep and man.

[56] Moradi MH, Nejati-Javaremi A, Moradi-Shahrbabak M, Dodds KG, McEwan JC (2012). Genomic scan of selective sweeps in thin and fat tail sheep breeds for identifying of candidate regions associated with fat deposition. BMC Genet.

[57] Lv FH, Peng WF, Yang J, Zhao YX, Li WR, Liu MJ (2015). Mitogenomic meta-analysis identifies two phases of migration in the history of eastern Eurasian sheep. Mol Biol Evol.

[58] Ryder ML (1981). A survey of European primitive breeds of sheep. Ann Genet Sel Anim..

[59] Dýrmundsson ÓR, Niżnikowski R (2010). North European short-tailed breeds of sheep: a review. Animal.

[60] Ivanov MF (1935). Ovtsevodstvo.

[61] Yunusbayev B, Metspalu M, Metspalu E, Valeev A, Litvinov S, Valiev R (2015). The genetic legacy of the expansion of Turkic-speaking nomads across Eurasia. PLoS Genet.

[62] Zhao YX, Yang J, Lv FH, Hu XJ, Xie XL, Zhang M (2017). Genomic reconstruction of the history of native sheep reveals the peopling patterns of nomads and the expansion of early pastoralism in East Asia. Mol Biol Evol.

[63] Gadzhiev ZK (2010). Grubosherstye ovtsy Dagestana.

[64] Musalaev K (2014). Sostoyanie I perspectivy razvitiya grubosherstnogo ovtsevodstva i kozovodstva Respubliki Dagestan. Sbornik nauchnykh trudov po materialam mezhdunarodnoi nauchno-prakticheskoi konferencii FGBNU VNIIOK.

[65] Tayshin VA, Lkhasaranov BB (1997). Aborigennaya buryatskasya ovtsa.

[66] Tayshin VA, Lkhasaranov VV, Shabanova RG (2001). Osnovnye prisnaki otbora aborigennyh buryatskikh. Ovtsy, kozy, sherstyanoe delo.

[67] Biltuev SI (2016). Sovremennoe sostoyanie polygrubosherstnogo i grubosherstnogo ovtsevodstva v Respublike Byryatia. Materialy Mezhdunarodnoi nauchno-prakticheskoi konferencii. posvyashennoi 60-letiu Zabaikal`skoi porody ovets.

[68] Averyanov IYA (1968). O proiskhozhdenii karakulskoy ovtsy. Ovtsevodstvo.

[69] Ivanov MF. Karakulevodstvo na uge Rossii: Opyt zootekh.-eccon. issled.Poltava: Izdatel`stvo Poltavskogo obtshestva sel`skogo khozyaystva; 1914 (**in Russian**).

[70] Ernst LK, Dmitriev NG, Paronyan IA (1994). Geneticheskie resursy sel`skokhozyaistvennykh zhivotnykh v Rossii i sopredel`nykh stranakh.

[71] Drăgănsecu C (1994). An attempt to a filetic classification of Valachian (Zackel) and Tsigai breed. Stocarstv.

[72] Drăgănsecu C (1995). Origin and relationships between Valachian and Tsigai sheep from the Danube area. Stocarstvo.

[73] Porter V, Alderson L, Hall SJG, Sponenberg DP (2016). Mason’s world encyclopedia of livestock breeds and breeding.

[74] Ilişiu E, Dărăban S, Radu R, Pădeanu I, Ilişiu VC, Pascal C (2013). The Romanian Tsigai sheep breed, their potential and the challenges for research. Appl Agric For Res.

[75] Ivanov MF (1929). Volosckie Ovta.

[76] Drăgănsecu C (2007). A note on Balkan sheep breeds origin and their taxonomy. Arch Zootech.

[77] Kosilov VI, Shkilev PN, Nikonova EA (2014). Produktivnye kachestva ovets raznykh porod na Uzhnom Urale.

[78] Medvedev MV, Erokhin AI (2004). Otkormocnye i uboinye kachestva ovets kuibyshevskoy porody i ee pomesei s myaso-sherstnymi baranami. Ovtsy, kozy, sherstyanoe delo.

[79] Kolosov Y (2014). Sal`skaya poroda ovets–istoria razvitiya i sovershenstvovanie. Sbornik nauchnykh trudov po materialam mezhdunarodnoi nauchno-prakticheskoi konferencii FGBNU VNIIOK.

[80] Egorov MV (2016). Sovremennoe sostoyanie ovtsevodstva v Rossiiskoi Federatsii. Mezhdunarodnoi nauchno-prakticheskoi konferencii. posvyashennoi 60-letiu Zabaikal`skoi porody ovets.

[81] Murzina TV, Vershinina VA (2016). Stanovlenie tonkorunnogo ovtsevodsrva i sovremennoe sostoyanie ovets v Zabaikal’skom krae. Informatsionnii bulleten.

