# Supplementary material for: Population structure and genetic diversity of 25 Russian sheep breeds based on whole-genome genotyping
Source: Genet Sel Evol. 2018 May 24;50:29. doi: 10.1186/s12711-018-0399-5 (PMC5968526; doi:10.1186/s12711-018-0399-5)
Supplement: Supplementary file 1 — Additional file 1: Table S1 Short description of the Russian sheep breeds under study. [file 12711_2018_399_MOESM1_ESM.pdf]

Breed	Code	Population size by year (thnd. heads)		Period of creation	Breeding region	History of creation, breeds involved in the development	Tail type	Production type
		2000	2015					
**COARSE WOOL SHEEP BREEDS**								
Andean Black	ANDB	15.4	170.0	14th–16th century	Mountain area of the Republic of Dagestan, North Caucasus	Breed was created by folk selection	Long fat	meat-wool-milk type
Buubei	BUUB	-	18.4	2008	Republic of Buryatia, Yakutia (Republic of Sakha)	Long-term improvement of indigenous Buryat sheep.	Short fat	meat-wool type
Edilbai	EDLB	14.5	163.0	19th century	Volgograd Region between the Ural River and the Volga River	Breed was raised by crossing Kazakh fat-rumped ewes with Astrakhan rams	Fat rump	meat-fat type
Kalmyk	KALM	29.7	27.3	unknown	Republic of Kalmykia	Originated from native fat-rumped sheep from China, which were improved with the Edilbai and Torgudsk breed,	Fat rump	meat-fat type
Karachaev	KRCH	46.7	301.8	14th–16th century	Mountain area of the Republic of Dagestan, North Caucasus	Breed was created by folk selection	Long fat	meat-wool-milk type
Karakul	KARA	28.1	41.4	unknown	Republic of Kalmykia, Astrakhan Region	Two main theories of creation:<br>1. Breed resulted from crossing the black indigenous sheep of Bukhara with Afghan and native fat-rumped sheep.<br>2. The ancestors of the Karakul were brought by	Long fat	sheepskin type

						Arabs to Middle Asia in the 8th century, where it was created.		
Kuchugur	KUCH	unknown	unknown	19th century	Voronezh and Kursk Areas	Mating local crossbred coarse wool ewes with large Voloshian rams	Long fat	meat-wool type
Lezgin	LEZG	41.3	127.0	14th–16th century	Mountain area of the Republic of Dagestan, North Caucasus	Breed was created by folk selection	Long fat	meat-wool-milk type
Romanov	RMNV	16.0	70.4	17th–18th century	Yaroslavl, Ryazan and Moscow Regions	The breed originated in the Yaroslavl region by long-term selection of local sheep	Short thin	skin coat and meat type
Tushin	TUSH	129.8	111.6	14th–16th century	Mountain area of the Republic of Dagestan, North Caucasus	Breed was created by folk selection	Long fat	meat-wool-milk type
Tuva	TUVA	61.3	241.1	unknown	Republic of Tyva	The breed was raised under the harsh climate by indigenous nomadic tribes	Short fat	meat-wool type
**SEMI-FINE WOOL SHEEP BREEDS**								
Altai Mountain	ALTM	159.7	84.9	1930-1940	Altai Region	The result of crossing local coarse wool fat-tailed sheep first with Grozny and then with Tsigai	Long thin	wool-meat type
Kuibyshev	KUIB	16.7	16.7	1936-1948	Samara Region	Mating local Cherkessk ewes with Romney rams; the F2 crosses were inbred without further use of Romney Marsh	Long thin	meat-wool type

North Caucasian	NCSN	52.1	20.8	1944-1961	Stavropol Region	Stavropol ewes were improved with Romney Marsh and Lincoln rams.	Long thin	meat-wool type
Russian Longhaired	RULH	47.0	12.3	1937-1978	Voronezh Area	Complex reproductive crossing of local coarse wool sheep (Kuchugur, Mikhnov and Northern short-tailed breeds) with British Lincoln	Long thin	meat-wool type
Tsigai	TZYG	203.1	45.9	800 BCE	Saratov and Rostov Regions	Originated in the Balkans and was brought to Russia by Transylvanian farmers from Romania in 1914	Long thin	wool-meat type
**FINE WOOL SHEEP BREEDS**								
Baikal fine-fleeced	BKFF	476.9	215.9	1927-1956	Yakutia (Republic of Sakha), Trans-Baikal area, Eastern Siberia	Improvement of local crossbred coarse wool Buryat-Mongolian sheep with Soviet Merino, Merino Précoce, Ascanian, Grozny and Altai rams	Long thin	wool-meat type

Dagestan Mountain	DAGM	435.3	845.5	1933-1950	Mountain and plain areas of the Republic of Dagestan, North Caucasus	Mating local coarse wool, fat-tailed ewes (Gunib breed) with rams of the Wurttemberg breed (Merinolandschaf).	Long thin	meat-wool type
Groznensk	GRZN	469.6	728.7	1923-1951	Republic of Kalmykia, Stavropol and Astrakhan Regions, the plains of the Republic of Dagestan, South of Russia, North Caucasus	Local fine wool New Caucasian and Mazaev ewes were improved with Australian Merino	Long thin	wool type
Kulundin	KLND	-	1.7	2008 (new type)	Altai Region	Introductory crossing of Altai ewes with Grozny, Australian Merino and Manych Merino rams	Long thin	wool-meat type
Manych Merino	MANM	45.6	26.4	1972-1983	Stavropol Region	Crossing of the Stavropol breed with Australian Merino	Long thin	wool type
Salsk	SALS	6.9	6.6	1922-1950	Rostov Region	Local New Caucasian and Mazaev ewes were improved with American Rambouillet	Long thin	wool type
Soviet Merino	SOVM	496.2	259.0	1920-1950	Stavropol Region	Local New Caucasian and Mazaev ewes were improved with Australian Merino and Rambouillet	Long thin	wool type
Stavropol	STAV	577.4	111.5	1923-1950	Stavropol Region	Local New Caucasian and Mazaev ewes were improved with Australian Merino and American Rambouillet	Long thin	wool type
Volgograd	VOLG	134.8	116.8	1946-1978	Volgograd Region	Mating local crossbred coarse wool, fat-tailed ewes	Long thin	meat-wool type

						with rams of different breeds (Merino Précoce, Caucasian, Grozny and New Caucasia)		
