# Supplementary material for: Population structure and genetic diversity of 25 Russian sheep breeds based on whole-genome genotyping
Source: Genet Sel Evol. 2018 May 24;50:29. doi: 10.1186/s12711-018-0399-5 (PMC5968526; doi:10.1186/s12711-018-0399-5)
Supplement: Supplementary file 2 — Additional file 2: Table S2 The joint dataset used in the study. Description: This table provides information on the joint dataset, which includes the Russian sheep breeds and sheep breeds from across the world. The breeds are grouped according to their ancestral geographic origin (Russia, the British Isles, Northern Europe, Central Europe, Southwestern Europe, Asia, Southwestern Asia, Africa and the Americas). The table presents the information concerning the breeds’ abbreviation and color representation for the geographical group of breeds, the sample size, country (region) of sample collection, and the references where the genotyping data were previously published. [file 12711_2018_399_MOESM2_ESM.pdf]

	Breed	Color / Code	n	Country (region) of sample collection (comments)	Source
	**Russia**				
	**Coarse wool breeds (CW)**				
1	Andean Black	ANDB	10	Republic of Dagestan	own
2	Buubei	BUUB	13	Yakutia (Republic of Sakha)	own
3	Edilbai	EDLB	14	Volgograd region	own
4	Kalmyk	KALM	16	Republic of Kalmykia	own
5	Karakul	KARA	20	Astrakhan region	own
6	Karachaev	KRCH	16	Karachaev-Cherkessk Republic	own
7	Kuchugur	KUCH	11	Voronezh region	own
8	Lezgin	LEZG	14	Republic of Dagestan	own
9	Romanov	RMNV	26	Yaroslavl region	own
10	Tushin	TUSH	9	Republic of Dagestan	own
11	Tuva	TUVA	16	Republic of Tyva	own
	**Semi-fine wool breeds (SFW)**				
12	Altai Mountain	ALTM	12	Russia, Altai region	own
13	Kuibyshev	KUIB	15	Russia, Samara region	own
14	North-Caucasian	NCSN	16	Russia, Stavropol region	own
15	Russian Longhaired	RULH	14	Russia, Voronezh region	own
16	Tsigai	TZYG	16	Russia, Saratov region	own
	**Fine wool breeds (FW)**				
17	Dagestan Mountain	DAGM	16	Russia, Republic of Dagestan	own
18	Groznensk	GRZN	13	Russia, Stavropol region	own
19	Kulundin	KLND	16	Russia, Altai region	own
20	Manych Merino	MANM	16	Russia, Stavropol region	own
21	Salsk	SALS	16	Russia, Rostov region	own
22	Soviet Merino	SOVM	14	Russia, Stavropol region	own
23	Stavropol	STAV	14	Russia, Stavropol region	own
24	Volgograd	VOLG	15	Russia, Volgograd region	own
	**British Isles**				
25	Australian Poll Dorset	APD	16	Australia	Kijas et al., 2012
26	Australian Suffolk	ASU	42	Australia (derived from English Suffolk)	Kijas et al., 2012
27	Badger Faced	BAF	16	Wales	Beynon et al., 2015
28	Brecknock Hill Cheviot	BHC	16	Wales	Beynon et al., 2015
29	Australian Coopworth	CPW	14	Australia (derived from NZ Coopworth)	Kijas et al., 2012
30	Galway	GAL	11	Ireland	Kijas et al., 2012
31	Hill Flock Welsh Mountain	HFWM	12	Wales	Beynon et al., 2015
32	Hill Radnor	HRA	12	Wales	Beynon et al., 2015
33	Improved Welsh Mountain	IWM	10	Wales	Beynon et al., 2015
34	Llandovery White Faced	LWF	18	Wales	Beynon et al., 2015
35	New Zealand Romney	ROM	17	New Zealand (derived from Engl. Romney)	Kijas et al., 2012

36	Scottish Blackface	SBF	18	Scotland	Kijas et al., 2012
37	Welsh Hardy Speckled Faced	WHSF	16	Wales	Beynon et al., 2015
	**Northern Europe**				
38	East Friesian Brown	EFB	10	Germany (originated from the Friesland)	Kijas et al., 2012
39	Finnsheep	FIN	44	Finland	Kijas et al., 2012
40	German Texel	GTX	22	Germany (derived from Dutch Texel)	Kijas et al., 2012
41	Norway Spaelsau	NSP	38	Norway	Kijas et al., 2012
42	New Zealand Texel	NTX	14	New Zealand (derived from Dutch Texel)	Kijas et al., 2012
	**Central Europe - Swiss**				
43	Bündner Oberländer Sheep	BOS	12	Switzerland	Kijas et al., 2012
44	Engadine Red Sheep	ERS	18	Switzerland	Kijas et al., 2012
45	Swiss Black-Brown Mountain	SBS	17	Switzerland	Kijas et al., 2012
46	Swiss Mirror Sheep	SMS	13	Switzerland	Kijas et al., 2012
47	Swiss White Alpine	SWA	18	Switzerland	Kijas et al., 2012
48	Valais Blacknose Sheep	VBS	12	Switzerland	Kijas et al., 2012
	**South-West Europe**				
49	Arawapa	APA	10	New Zealand	Ciani et al., 2015
50	Australian Poll Merino	APM	46	Australia (derived from Spanish Merino)	Kijas et al., 2012
51	Appenninica	APP	22	Italy	Ciani et al., 2014
52	Castellana	CAS	17	Spain	Kijas et al., 2012
53	Chios	CHI	16	Greece	Kijas et al., 2012
54	Churra	CHU	50	Spain	Kijas et al., 2012
55	Chinese Merino	CME	22	China (derived from Spanish Merino)	Kijas et al., 2012
56	Comisana	COM	24	Italy	Ciani et al., 2014
57	Gentile di Puglia	GEN	18	Italy	Ciani et al., 2014
58	Lacaune	LAC	43	France	Kijas,2012
59	Laticauda	LAT	21	Italy	Ciani et al., 2014
60	Leccese	LEC	17	Italy	Ciani et al., 2014
61	Massese	MAS	14	Italy	Ciani et al., 2014
62	Merino	MER	48	Spain	Kijas et al., 2012
63	Merinolandschaf	MLA	20	Germany (derived from Spanish Merino)	Kijas et al., 2012
64	Merinizzata Italiana	MNZ	17	Italy	Ciani et al., 2015
65	Ojalada	OJA	24	Spain	Kijas et al., 2012
66	Rasa Aragonesa	RAA	19	Spain	Kijas et al., 2012
67	Rambouillet	RMB	40	France	Kijas et al., 2012
68	Sardinian White	SAW	19	Italy	Kijas et al., 2012
69	Spanish Merinos Estrema Dura	SMED	13	Spain	Ciani et al., 2015
70	Sopravissana	SOP	22	Italy	Ciani et al., 2014
	**Asia**				
71	Changthangi	CHA	16	China (Tibet)	Kijas et al., 2012
72	Deccani	IDC	16	India	Kijas,2012
73	Tibetan	TIB	14	China (Tibet)	Kijas et al., 2012

	**South-West Asia**				
74	Afshari	AFS	19	Iran	Kijas et al., 2012
75	Moghani	MOG	29	Iran	Kijas et al., 2012
76	Qezel	QEZ	32	Iran	Kijas et al., 2012
	**South Africa**				
77	African Dorper	ADP	10	South Africa	Kijas et al., 2012
78	Ethiopian Menz	EMZ	21	Ethiopia	Kijas et al., 2012
	**Americas**				
79	Barbados Black Belly	BBB	14	Barbados	Kijas et al., 2012
80	Brazilian Creole	BCS	15	Brazil	Kijas et al., 2012
81	Santa Ines	BSI	28	Brazil	Kijas et al., 2012
82	Gulf Coast Native	GCN	40	USA	Kijas et al., 2012
