# Supplementary material for: Population structure and genetic diversity of 25 Russian sheep breeds based on whole-genome genotyping
Source: Genet Sel Evol. 2018 May 24;50:29. doi: 10.1186/s12711-018-0399-5 (PMC5968526; doi:10.1186/s12711-018-0399-5)
Supplement: Supplementary file 3 — Additional file 3: Table S3 Genetic differentiation of 25 Russian sheep breeds based on Weir and Cockerham’s fixation index (FST). This table provides information about FST values between the Russian breeds under study. The breeds’ groups of the same wool type are framed in blue (for coarse wool breeds), red (for semi-fine wool breeds) and green (for fine wool breeds). For a description of the sheep breeds (see Additional file 1: Table S1, Additional file 2: Table S2). [file 12711_2018_399_MOESM3_ESM.pdf]

Breed codes	Fixation index (Fst)																									
	ANDB	BUUB	EDLB	KALM	KRCH	KARA	KUCH	LEZG	RMNV	TUSH	TUVA	ALTM	KUIB	NCSN	RULH	TZYG	BKFF	DAGM	GRZN	KLND	MANI	SALS	SOVM	STAV	VOLG
ANDB																									
BUUB	0,064																								
EDLB	0,047	0,038																							
KALM	0,046	0,038	0,007																						
KRCH	0,036	0,042	0,027	0,027																					
KARA	0,049	0,043	0,023	0,024	0,031																				
KUCH	0,103	0,095	0,085	0,082	0,079	0,089																			
LEZG	0,040	0,043	0,026	0,025	0,023	0,028	0,082																		
RMNV	0,113	0,104	0,096	0,093	0,088	0,101	0,124	0,095																	
TUSH	0,038	0,043	0,026	0,025	0,021	0,027	0,083	0,018	0,095																
TUVA	0,045	0,033	0,018	0,018	0,025	0,024	0,083	0,026	0,092	0,025															
ALTM	0,064	0,055	0,046	0,043	0,033	0,049	0,073	0,040	0,085	0,038	0,044														
KUIB	0,077	0,066	0,059	0,055	0,048	0,062	0,077	0,052	0,084	0,052	0,056	0,030													
NCSN	0,096	0,084	0,078	0,073	0,064	0,081	0,091	0,070	0,100	0,069	0,076	0,041	0,020												
RULH	0,105	0,096	0,088	0,085	0,075	0,092	0,100	0,083	0,112	0,081	0,086	0,059	0,046	0,048											
TZYG	0,062	0,054	0,044	0,041	0,035	0,046	0,068	0,037	0,085	0,036	0,042	0,013	0,030	0,044	0,059										
BKFF	0,068	0,053	0,047	0,042	0,036	0,050	0,075	0,039	0,085	0,039	0,043	0,018	0,020	0,027	0,057	0,020									
DAGM	0,070	0,063	0,055	0,051	0,044	0,057	0,082	0,044	0,091	0,043	0,053	0,031	0,038	0,047	0,070	0,031	0,022								
GRZN	0,075	0,063	0,058	0,052	0,045	0,060	0,079	0,047	0,088	0,046	0,055	0,022	0,026	0,031	0,062	0,023	0,006	0,024							
KLND	0,098	0,085	0,080	0,074	0,068	0,082	0,100	0,071	0,110	0,071	0,077	0,043	0,048	0,052	0,084	0,045	0,027	0,045	0,024						
MANM	0,082	0,071	0,065	0,059	0,053	0,068	0,086	0,055	0,095	0,055	0,062	0,029	0,034	0,039	0,070	0,030	0,015	0,032	0,010	0,028					
SALS	0,086	0,072	0,067	0,062	0,055	0,070	0,087	0,057	0,097	0,057	0,065	0,030	0,034	0,038	0,072	0,032	0,014	0,033	0,011	0,029	0,018				
SOVM	0,080	0,065	0,061	0,055	0,049	0,064	0,081	0,051	0,092	0,050	0,058	0,024	0,029	0,034	0,066	0,025	0,007	0,027	0,004	0,023	0,011	0,008			
STAV	0,085	0,072	0,067	0,062	0,054	0,070	0,087	0,057	0,097	0,057	0,064	0,030	0,034	0,038	0,071	0,032	0,014	0,032	0,008	0,028	0,012	0,014	0,008		
VOLG	0,086	0,073	0,067	0,062	0,055	0,071	0,090	0,058	0,100	0,058	0,065	0,034	0,039	0,044	0,074	0,035	0,018	0,036	0,018	0,036	0,027	0,023	0,016	0,024	
