# Supplementary figures and images for: Population structure and genetic diversity of 25 Russian sheep breeds based on whole-genome genotyping
Source: Genet Sel Evol. 2018 May 24;50:29. doi: 10.1186/s12711-018-0399-5 (PMC5968526; doi:10.1186/s12711-018-0399-5)

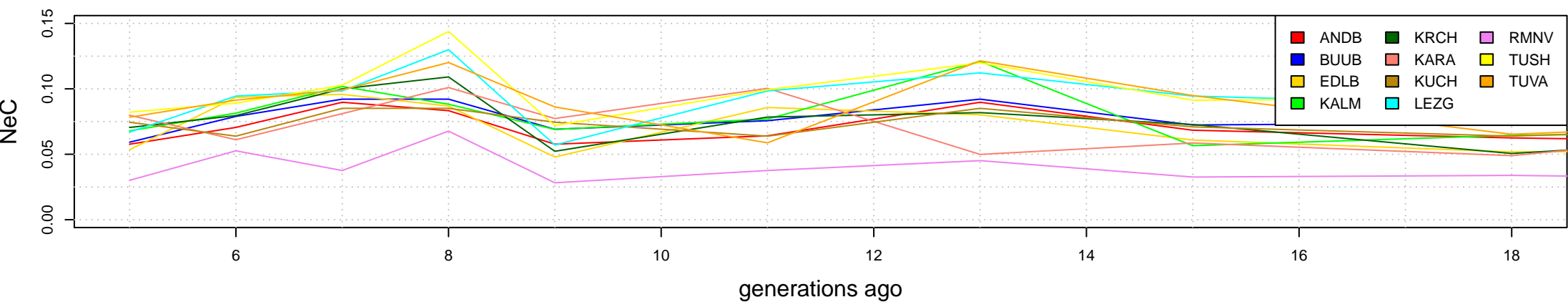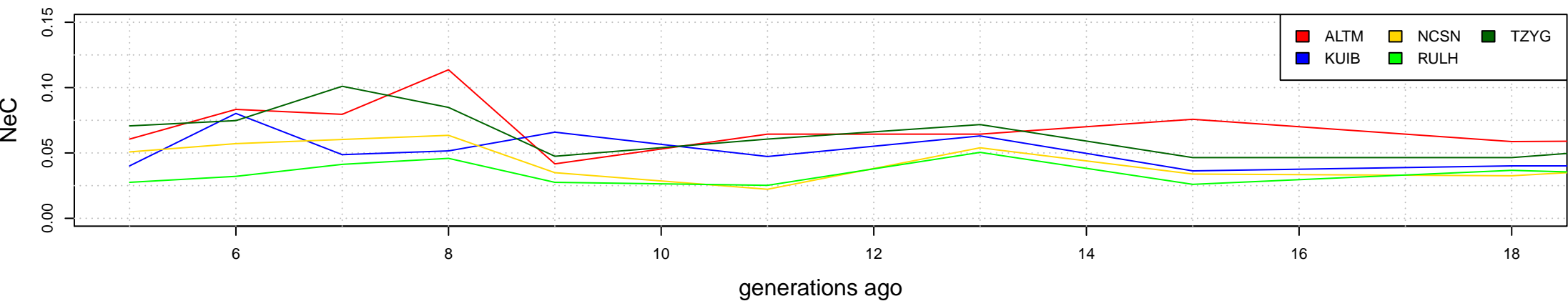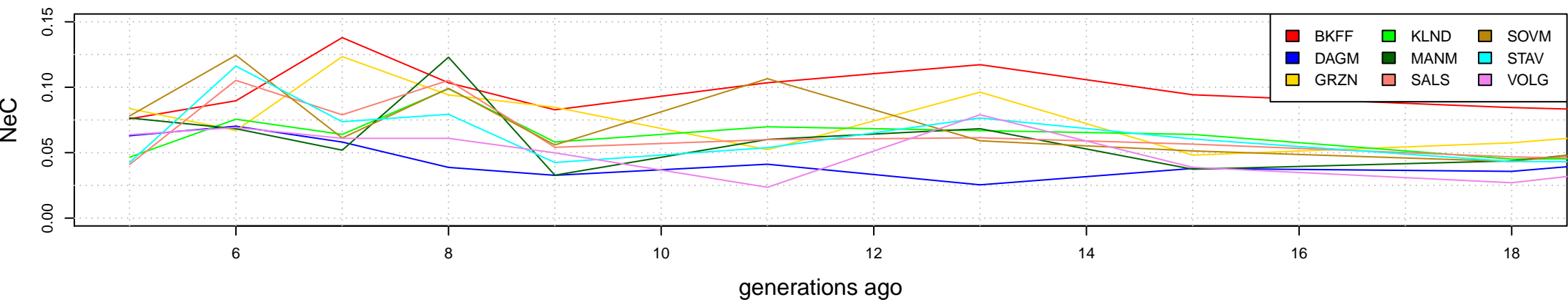

Supplement: Supplementary file 4 — Additional file 4: Figure S1 Slope changes in historical effective population size (Ne) trends. The graphs show the changes in slope trends for historical effective population size (Ne) for the period starting from approximately 18 generations ago for the Russian sheep breeds with the coarse wool (above), semi-fine wool (in the middle) and fine wool (below). For a description of the sheep breeds (see Additional file 1: Table S1, Additional file 2: Table S2). [file 12711_2018_399_MOESM4_ESM.pdf]
